# Thermal-field coupling toward efficient water electrolysis

**DOI:** 10.3389/fchem.2026.1888564

**Published:** 2026-07-27

**Authors:** Duanduan Liu, Jiayi Wan, Shuo Gao, Hongye Li

**Affiliations:** 1 College of Electronic and Engineering, Nanjing Xiaozhuang University, Nanjing, Jiangsu, China; 2 Eco-materials and Renewable Energy Research Center (ERERC), Collaborative Innovation Center of Advanced Microstructures, College of Engineering and Applied Sciences, Nanjing University, Nanjing, Jiangsu, China

**Keywords:** electronic state regulation, oxygen evolution reaction, thermo-electricity coupling, waste heat, water electrolysis

## Abstract

Electrochemical water splitting is limited by sluggish oxygen evolution kinetics and high energy consumption. Thermo-electricity coupling, utilizing heat and electric fields, has become an effective strategy to break away from traditional thermal methods and improve electrocatalytic efficiency. This work summarizes the core mechanisms of thermo-electric coupled water splitting, including thermal suppression of charge disproportionation, thermal driven spin regulation and thermal strain engineering, while elucidated the thermal effects beyond mass transfer. Utilizing industrial or power waste heat as a low-cost heat source, this strategy improved energy efficiency and matched with renewable energy systems. Besides, the challenges and prospects are proposed for the practical development of high-efficiency thermo-electricity coupled hydrogen production.

## 1 Introduction

As the world accelerates the transition from fossil fuels to renewable energy systems, green hydrogen produced by electrochemical water splitting has become the critical carrier of low-carbon energy strategies owing to its high energy density, clean combustion products and versatile storage capabilities (Gao et al., 2024; Jamesh et al., 2024; Kazemi et al., 2024; McCrory et al., 2013; Xiong and He, 2023). Among existing technologies, alkaline water and proton-exchange membrane water electrolysis represent the most mature routes, but both suffer from inherent bottleneck. The anodic oxygen evolution reaction (OER) involves four proton-coupled electron-transfer steps and multiple adsorbed oxygen intermediates, resulting in large overpotentials, low electricity-to-hydrogen efficiencies, and heavy reliance on high-performance electrocatalysts (Matienzo et al., 2020; Suen et al., 2017). These bottlenecks highlight the urgent need to develop high-performance electrocatalysts under harsh working environments to facilitate scalable industrial deployment (Ul Sahar et al., 2026). Over the past decades, tremendous efforts have been devoted to catalyst design, including elemental doping (Fan et al., 2024; Zhang Y. Q. et al., 2025), defect engineering (Yan et al., 2023; Zhang Y. Q. et al., 2025), strain modulation (Hou et al., 2023; Ma et al., 2023), and interface engineering (Guo et al., 2021), aiming to optimize the adsorption-desorption energetics of reaction intermediates and accelerate reaction kinetics. To address the low catalytic activity restricted by OER kinetics, a wide range of material modification strategies have been proposed in electrocatalysis and carbon-neutral conversion research, covering flexible textile-based sulfide electrocatalysts (Zhou et al., 2024), MOF-derived phosphide catalysts (Guo et al., 2023), ligand-tuned pre-strain MOF catalysts (Ou et al., 2025), noble metal cluster electronic modulation of layered double hydroxides (Li et al., 2026), bimetallic oxide-carbon composite bifunctional electrocatalysts (Qaseem et al., 2026), spinel ferrite piezophotoelectrocatalysts (Jabeen et al., 2025) and Z-scheme piezo-photocatalytic composite systems (Mumtaz et al., 2024). Meanwhile, catalytic conversion routes for CO_2_ upgrading to sustainable aviation fuels have also been developed to advance carbon neutralization (Tian et al., 2024). However, due to the involvement of complex intermediates in OER, it is difficult to break the inherent scaling relationships between the adsorption energies of intermediates, which severely limits researchers to regulate the catalytic energy barrier (Seh et al., 2017; Zhang Z. Y. et al., 2025).

Utilizing the waste heat released from industrial production and geothermal processes to produce hydrogen is an efficient way to utilize energy and reduce dependence on fossil fuels (Abraham et al., 2013). Thermo-electric coupling water splitting introduces thermal energy as an external energy field and drives the electrolysis process together with electrical energy. The traditional heating only accelerates the material transfer process and follows the linear Arrhenius relationship, while the thermo-electric coupling triggers intrinsic heatphysical effects of material, dynamically reconfiguring the electronic state, spin configuration and local lattice environment (Hedayati et al., 2026). These changes fundamentally lower interfacial electron-transfer barriers and induces non-linear enhancements of activity which cannot be achieved by electricity or heat alone. Moreover, this strategy enables the efficient utilization of waste heat, industrial exhaust heat or solar thermal energy, greatly improving the overall energy efficiency and compatibility with renewable power system.

In this review, we summarized the fundamental mechanisms of thermo-electricity coupling water splitting, which involved in thermal regulation of charge disproportionation, spin states and lattice strain. We highlighted representative catalyst systems and structural designs, and clarify the synergistic interactions between thermal and electric fields. Furthermore, we also summarized the critical technical challenges and perspectives faced in promoting the practical application of thermo-electric coupling technology.

## 2 Physical mechanism of thermo-electricity coupling water splitting

**FIGURE 1:**
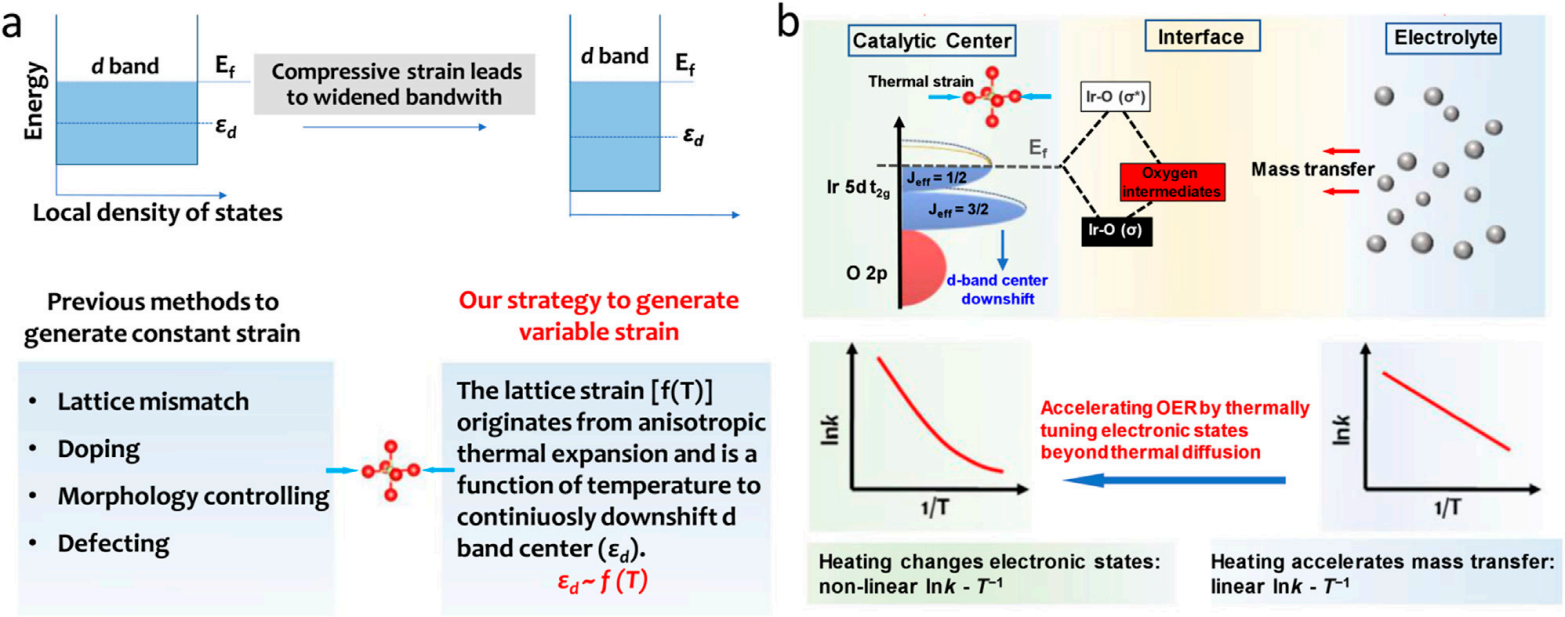
**(a)** Schematic illustration of compressive strain effects. Heating simultaneously accelerates mass transport and generates compressive strain in IrO_6_ octahedra, which downshifts the d-band center. Reproduced with permission from Du et al. (2024). Copyright 2024 Springer Nature. **(b)** Mechanistic overview of thermally enhanced OER kinetics. In Sr_2_IrO_4_, strong spin-orbit coupling of low-spin Ir^4+^ (5 d^5^, t_2g_^5^eg_0_) splits the t_2g_ band into *J*_eff_ = 3/2 sub-band and *J*_eff_ = 1/2 sub-band. The thermally induced strain and d-band shift are temperature-dependent and reversible, in which the thermal diffusion followed linear Arrhenius behavior. But the strain-governed electrocatalysis exhibits nonlinear kinetics process by optimizing Ir-O bonding and antibonding states to satisfy the Sabatier principle. Reproduced with permission from Du et al. (2024). Copyright 2024 Springer Nature.

Different from traditional Arrhenius-type thermal acceleration, the thermo-electric coupling effect has an inherent regulatory effect on electron spin state and lattice structure of electrocatalysts, providing a flexible strategy for breaking through the kinetic limitations of oxygen evolution reactions. As shown in Figure 1, a typical mechanism is thermally induced strain engineering, which dynamically optimizes the center position of d orbitals through anisotropic lattice expansion. For example, moderate heating can induce uniaxial compressive strain inside the IrO_6_ octahedron in Sr_2_IrO_4_, shortening the Ir-O bond length and enhancing metal oxygen covalency, thereby lowering the center position of *d* orbital (Du et al., 2024). This strategy optimizes the adsorption desorption strength of intermediates, reduces the energy barrier and achieves non-linear activity enhancement, accordingly different from simply accelerating mass transfer. Unlike the strain drived by lattice mismatch or doping, the thermally induced strain is reversible and can be adjusted by temperature, enabling real-time electronic structure control under operating conditions.

**FIGURE 2:**
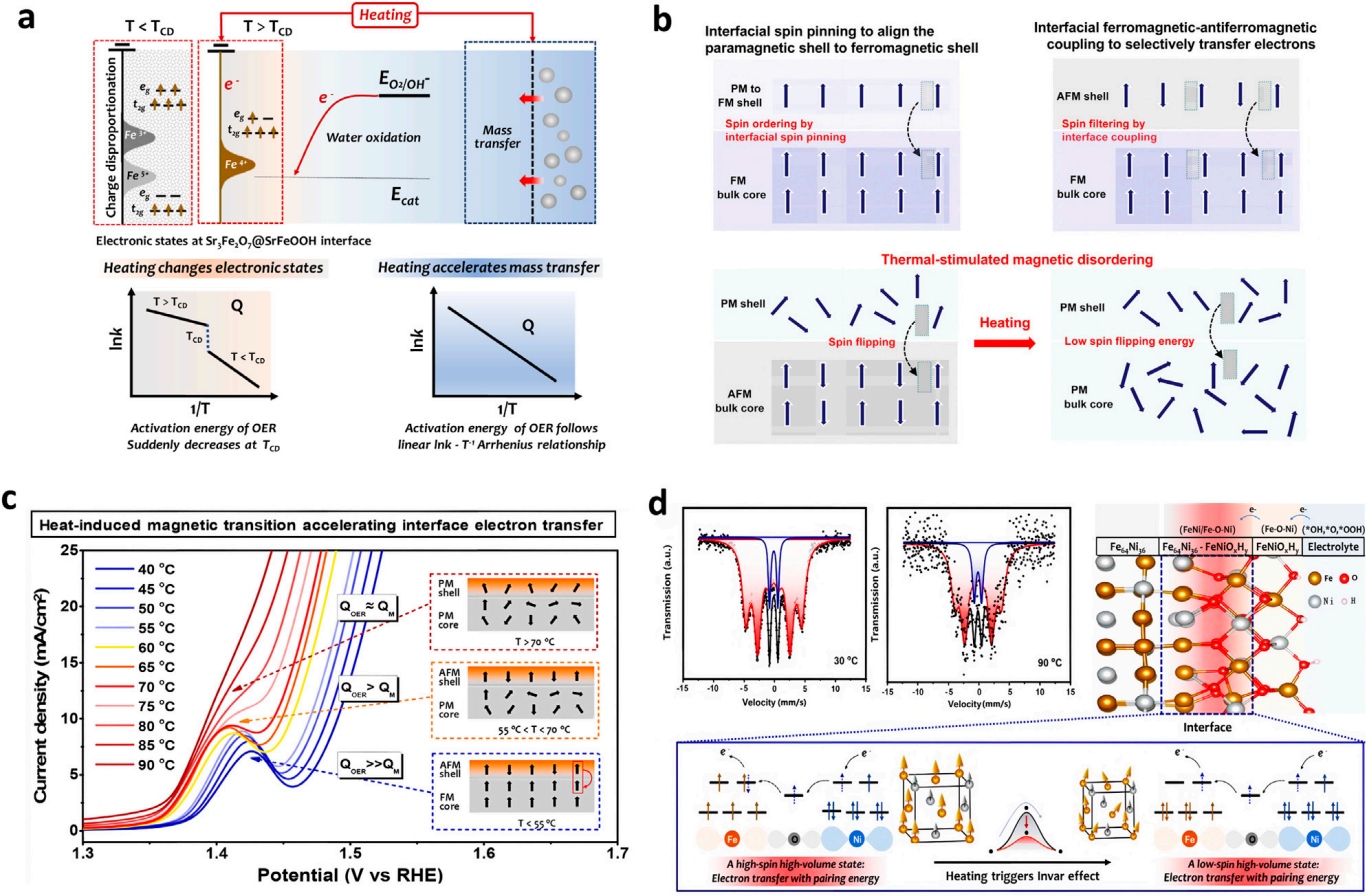
**(a)** A scheme to describe accelerating OER by thermally inhibited CD beyond thermal diffusion. Coupling heat to inhibit CD would provide more unoccupied orbitals for interface electron transfer. The slope of linear ln(k) vs. 1/T Arrhenius relationship at a given temperature to trigger thermophysical effect of materials was changed. Reproduced with permission from Lu et al. (2024b). Copyright 2023 National Academy of Sciences, U.S.A. **(b)** Interface spin pinning between the ferromagnetic core and paramagnetic shell induces paramagnetic-to-ferromagnetic transition of the catalytic shell to produce a spin-ordering interface amd Interface ferromagnetic-antiferromagnetic coupling facilitates the selective removal of electrons with spin direction opposing the magnetic moment of the ferromagnetic core. Reproduced with permission from Xie et al. (2025). Copyright 2025 Royal Society of Chemistry. **(c)** The temperature-dependent spin-related LSV curves. Reproduced with permission from Lu et al. (2022). Copyright 2022 American Chemical Society. **(d)** The mechanism for accelerating electron transfer by thermal Invar effect of Fe_64_Ni_36_/FeNiO_x_H_y_. Reproduced with permission from Lu et al. (2024a). Copyright 2024 American Physical Society.

The thermal field could effectively reconfigure interfacial spin configurations to eliminate spin-flip barriers and accelerate charge transfer in magnetic heterostructures. For example, the FeNiO_x_H_y_ undergoes a ferrimagnetic-to-paramagnetic transition of Curie temperature occurring about 70 °C, which corresponding to a second-order Curie-type magnetic transition. This transition has generated low-spin Fe^3+^ species with enhanced orbital availability for electron hopping, accomplished the paramagnetic Ni^2+^/Ni^3+^ oxidation and Ni^3+^ oxidation of water, effectively reduced the electron transfer barrier and delivered 100 mA cm^-2^ at an overpotential of 221 mV at 90 °C^28^. In NiFeN@NiFeOOH core-shell catalysts, the thermal stimulation at 55 °C–70 °C in Figure 2c drives catalyst to paramagnetic states, eliminating ferromagnetic-antiferromagnetic spin friction and forming a spin-aligned interface for unimpeded charge transport (Lu et al., 2022). Similarly, the thermal excitation triggered the transformation of Fe atoms in the Fe_64_Ni_36_ invar alloy from a high-spin to a low-spin configuration in 3*d* orbitals by invar effect, offering more unoccupied orbitals as electron-transfer pathways at the Fe_64_Ni_36_/FeNiO_x_H_y_ interface. This effect greatly accelerates interfacial electron transfer and boosts the water oxidation performance of Fe_64_Ni_36_/FeNiO_x_H_y_ catalyst (Lu et al., 2024a) (Figure 2d). Meanwhile, taking YFe_1-x_M_x_O_3_ as a typical catalyst, the thermal excitation triggers its antiferromagnetic-to-paramagnetic phase transition, generating a magnetically disordered interface in YFe_1-x_M_x_O_3_@YFeOOH. This effectively reduces the spin-flip barrier for interfacial electron transfer. The strong interfacial interaction between YFe_1-x_M_x_O_3_ and the YFeOOH catalytic layer, inducing a highly active electronic state in YFeOOH (Figure 2b). Consequently, the linear Arrhenius relationship is broken, which enabled a pronounced thermal-electricity coupled catalytic effect (Xie et al., 2025). Other critical mechanism is that the heat could suppress the charge disproportionation (CD) of Fe^4+^ to Fe^3+^ and Fe^5+^ in Sr_3_Fe_2_O_7_ at T_CD_ of 67 °C. In Figure 2a, the charge disproportionation of 2Fe^4+^→Fe^3+^+Fe^5+^ is persistd at T < 67 °C and is fully thermally suppressed at T > 67 °C to stabilizing high-spin Fe^4+^ (t_2g_^3^e_g_^1^) with available orbitals as electron transfer channels, thereby accelerating interfacial electron transfer across the bulk/catalytic layer interface of Sr_3_Fe_2_O_7_@SrFeOOH. In this process, the relationship of temperature and reaction rate is deviated from the linear Arrhenius behavior, which confirmed that heat modulates the electronic states of the catalyst and thus intrinsically boosts catalytic activity (Lu et al., 2024b).

**FIGURE 3:**
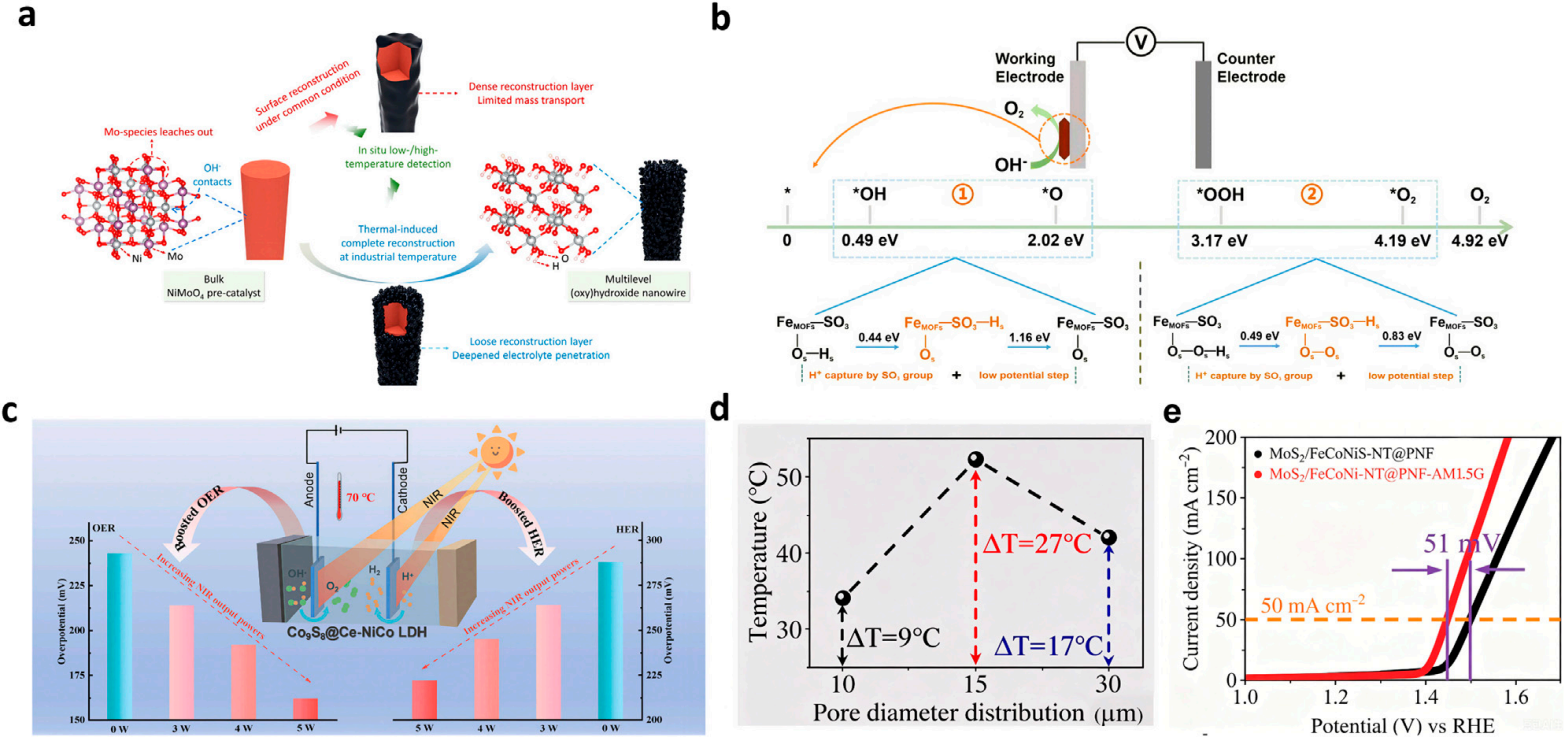
**(a)** The reconstruction of NiMoO_4_ pre-catalyst under low- and high-temperature electro-oxidation conditions. Reproduced with permission from Liu et al. (2020). Copyright 2020 John Wiley and Sons. **(b)** The thermocatalytic process cascaded electrocatalytic pathway of Fe_MOFs_-SO_3_ catalyst. Reproduced with permission from Feng et al. (2020). Copyright 2020 John Wiley and Sons. **(c)** NIR photothermal boosted OER/HER catalysis over Co_9_S_8_@Ce-NiCo LDH heterostructure electrolyzer. Reproduced with permission from Yang et al. (2024). Copyright 2024 Elsevier. **(d)** The temperature of different amount MoS_2_/FeCoNiS-nanotube@porous nickel foam electrode with AM 1.5G, and **(e)** LSV polarization curves of MoS_2_/FeCoNiS-nanotube@porous nickel foam electrodes without and with AM1.5G. Reproduced with permission from Zhou et al. (2023). Copyright 2023 John Wiley and Sons.

Furthermore, as shown in Figure 3a, thermal fields can drive beneficial surface reconstruction to form durable active phases. For example, the moderate heating could promote the *in-situ* transformation of precatalysts into oxyhydroxide layers rich in defects and grain boundaries, which lower OER energy barriers while enhancing structural robustness. The thermally stabilized reconstructed catalysts possess a low overpotentials of 282.3 mV at 20 mA cm^-2^ for over 250 h, demonstrating the synergy between thermal activation and long-term stability for industrial operations (Liu et al., 2020). Collectively, these thermal mechanisms including strain tuning, spin reconfiguration, charge-state stabilization and structural evolution has established thermal field coupling, which could conducted as a powerful approach to transcend conventional catalyst design rules.

Beyond intrinsic electronic and structural effects, the photothermal effect provides an additional pathway for localized heating (Figures 3c–e). Co_9_S_8_@Ce-NiCo LDH/NF heterostructures reached 62.5 °C under near-infrared (NIR), enhancing HER by 24.6% and OER by 15.8% (Yang et al., 2024). The photothermal effect also lowered the HER activation energy in alkaline seawater from 29.7 to 18.4 kJ mol^-1^ for NiMo-H_2_ catalysts under one sun (Wu et al., 2025). A leaf-structure-inspired through-hole electrode decorated with MoS_2_/FeCoNiS nanotubes achieved a temperature rise from 25 °C to 52.3 °C, reducing the OER overpotential (Figure 3e) (Zhou et al., 2023). Similarly, RuNiFeO_x/_FeNi_3_ heterostructured arrays with Janus wettability promoted electrolyte contact and gas bubble release. Under NIR light, the surface temperature increased to 44.3 °C, and the OER overpotential decreased from 253 to 213 mV at 50 mA cm^-2^ (Ai et al., 2024).

To characterize the thermally modulated electronic, magnetic and structural transformations occurring during thermally coupled water electrolysis, the variable-temperature operando and advanced characterization approaches by treating the samples at different temperatures have been summarized in this work. Variable-temperature operando XPS and Mössbauer spectroscopy could track temperature-dependent metal valence evolution and validate thermally driven charge comproportionation behavior. *In-situ* XRD and *in-situ* Raman spectroscopy are powerful tools to capture thermally activated lattice distortion, octahedral rotation and adjustable lattice strain in catalyst lattices. For probing thermally modulated spin states and magnetic phase transitions, the EPR and variable-temperature SQUID measurements are applicable to determine critical magnetic transition temperatures and spin disorder trends, while the XANES further distinguishes high/low-spin configurations of metal active centers. In addition, temperature-resolved operando EIS, open-circuit potential attenuation measurements and *in-situ* UV-Vis spectroscopy are combined to dynamically record interfacial electron transfer kinetics and redox species evolution synchronously with thermal field variation. These advanced characterization techniques provide a great experimental platform to separately resolve temperature-dependent structural, magnetic and electronic changes during electrolytic water splitting.

## 3 Thermo-electricity coupling strategies for efficient electrocatalytic water splitting: cascade, integration and multifunctional hybrid

**FIGURE 4:**
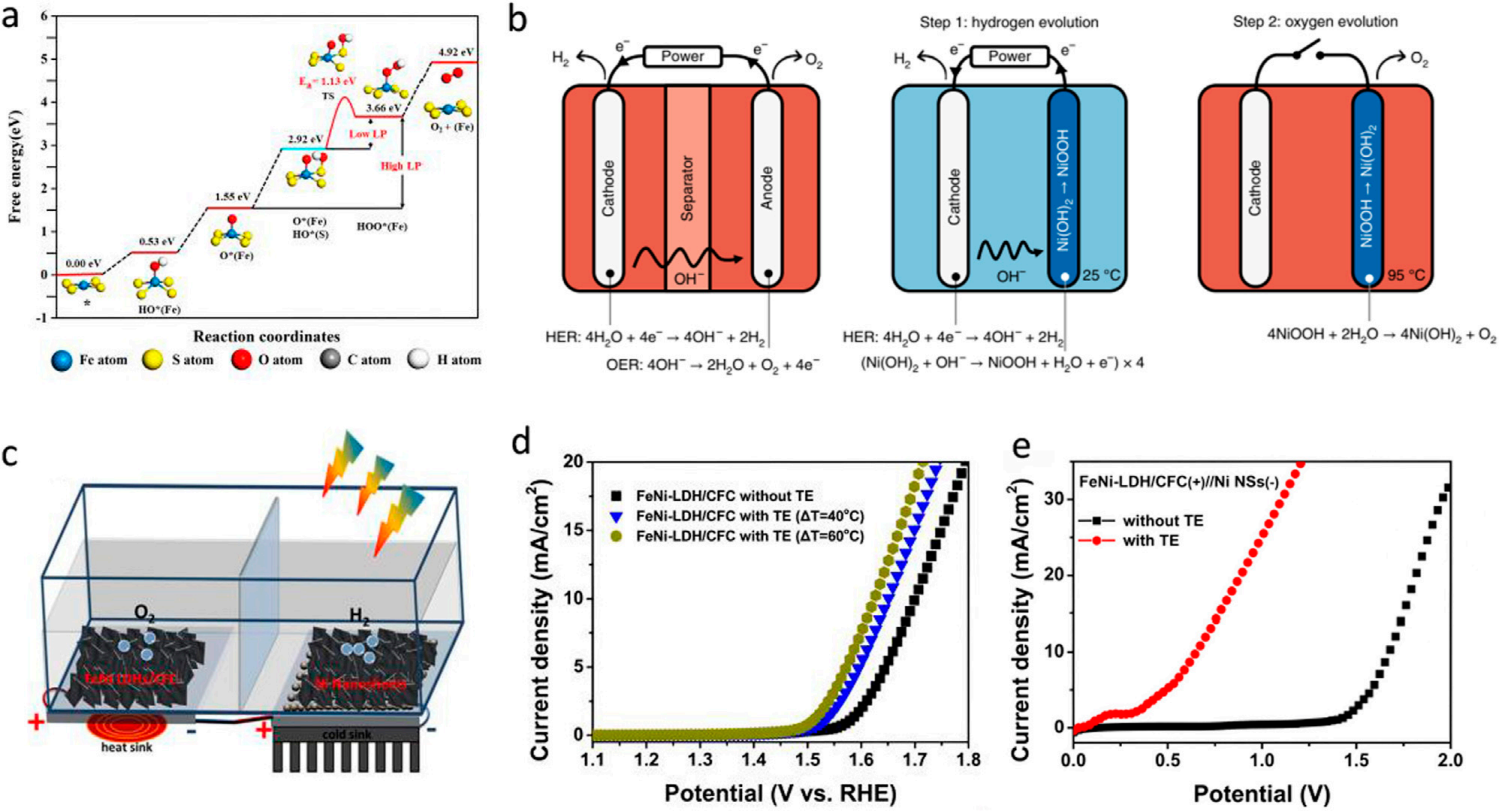
**(a)** Energy profile of thermally and electrically cascaded OER pathway on a Fe-BHT monolayer. Reproduced with permission from Fan et al. (2022). Copyright 2022 American Chemical Society. **(b)** Schematic of alkaline water electrolysis and the E-TAC water-splitting process. An electrochemical step reduces water by the conventional HER at the cathode, liberating OH^−^ that oxidize Ni(OH)_2_ anode into NiOOH, following by a chemical step, wherein the NiOOH anode reacts with water to spontaneously produce oxygen. Reproduced with permission from Dotan et al. (2019). Copyright 2019 Springer Nature. **(c)** Illustration of Electrolyzer-TE hybrid device, **(d)** polarization curves for OER, and **(e)** polarization curves of the alkaline Electrolyzer-TE hybrid device using Ni nanosheets as the cathode and NiFe-LDH/CFC as the anode with TE and without TE. Reproduced with permission from Zhao et al. (2019). Copyright 2019 Elsevier.

Beyond intrinsic thermal regulation of catalyst electronic, spin, and lattice structures, the thermo-electricity coupling has enables a cascaded reaction pathway which bypassed the conventional scaling relations. In Fe-benzenehexathiol (Fe-BHT) coordination polymers, the thermal energy has assisted the coupling of *O on Fe sites and *OH on adjacent S atoms, forming *OOH via a thermally activated route rather than the traditional high-barrier single-site mechanism (Figure 4a). Thermo-electrocatalytic cascade process successfully reduced the potential-limiting step energy, delivering an overpotential of 282 mV at 10 mA cm^-2^ and 80 °C, which outperforms commercial IrO_2_ under identical conditions. The cascaded mechanism is distinguished by a steeply decreasing Tafel slope with rising temperature and confirmed a qualitative change in kinetics beyond simple thermal acceleration (Fan et al., 2022). Complementary to catalytic cascades, the thermo-electricy interface integration further extends the thermal utilization to overall water splitting. Graphene oxide (GO) coatings enhance infrared absorption and photothermal conversion on commercial thermoelectric devices, hence creating localized thermal gradients that boost output voltage. When coupled with Bi_2_WO_6_ photoelectrodes, the thermoelectric potential provides a self-sustained bias that suppresses electron-hole recombination, increasing photocurrent by 5.6 times and doubling phenol degradation efficiency under solar irradiation (Sun et al., 2014). This architecture could converts low-grade thermal energy directly into a useful electrocatalytic bias, effectively offering a scalable route to recover waste heat for energy-saving water electrolysis. Further advancing this concept, multifunctional electrocatalyst-thermoelectric hybrid systems have been developed. As shown in Figure 4c, Ni nanosheet arrays are integrated on the hot side of thermoelectric devices serve simultaneously as photothermal converters and as electrocatalysts for the hydrogen evolution reaction. These porous Ni nanosheets produced strong localized heating under sunlight, thereby creating a large temperature difference (ΔT) to drive thermoelectric voltage output. Figures 4d,e showed that the introduction of thermoelectric module can increase the temperature difference. After integrating the thermoelectric coupling system, the voltage of electrolysis system at the same current density is greatly reduced. This dual-functional design unifies heat harvesting, charge generation and electrocatalysis have enabled efficient utilization of solar thermal energy and industrial waste heat for sustainable water splitting (Zhao et al., 2019). Sulfur-treated Fe-based MOFs (Fe-MOF-SO_3_) further validated the thermo-electrocatalytic cascade approach to oxygen evolution. The -SO_3_ groups acted as thermally responsive proton-capturing sites, which can extracting H^+^ from *OH/*OOH intermediates on Fe active centers to lower the potential-limiting barrier, thereby delivered a low overpotential of 218 mV at 10 mA cm^-2^ and 296 mV at 1000 mA cm^-2^ at 50 °C, as well as a excellent stability for industrial water electrolysis. *In-situ* spectra and DFT verified the S-O-Fe distortion and thermally assisted proton transfer could act as the core origin of boosted OER kinetics (Feng et al., 2020) (Figure 3b). Beyond cascade catalysis and interface integration, the electrochemical-thermally activated chemical (E-TAC) cycle has established a thermo-electrically decoupled water-splitting system. As shown in Figure 4b, in this system, the HER proceeded at room temperature to oxidize Ni_0.9_Co_0.1_(OH)_2_ anodes without O_2_ generation, while the hot alkaline electrolyte (95 °C) triggered spontaneous O_2_ release and anode regeneration. The OER catalytic performance of diverse catalysts under pure electrical and thermo-electric coupling conditions is summarized in Table 1. The membrane-free process effectively avoiding H_2_/O_2_ crossover and reducing electrochemical polarization via thermal input, providing a high-efficiency, scalable platform for heat-assisted overall water splitting (Dotan et al., 2019). Although the membrane-free E-TAC suppresses gas crossover and cuts hardware costs, the anode heating at 95 °C for spontaneous O_2_ evolution creates extra heat loss and reduces system efficiency. Nevertheless, quantitative energy balance manifest that the additional energy consumption limited to less than 3 kWh per kg of produced H_2_.

**TABLE 1:** Comparison of OER catalytic performance under pure electrical and thermo-electric coupling conditions for various catalysts.

Catalyst	Temperature (^o^C)	Overpotential (mV)	Tafel slope (mVdec^−1^)	Test condition	Ref.
Fe-BHT	20 (Pure Electric)	372@10 mA cm^-2^	151.7	1.0 M KOH	Fan et al. (2022)
	80 (Thermoelectric)	282@10 mA cm^-2^	33.5	1.0 M KOH	Fan et al. (2022)
Sr_2_IrO_4_	20 (Pure Electric)	312@10 mA cm^-2^	90.32	1.0 M KOH	Du et al. (2024)
	90 (Thermoelectric)	235@10 mA cm^-2^	45.14	1.0 M KOH	Du et al. (2024)
YFe_0.6_Mn_0.4_O_3_@YFeOOH	30 (Pure Electric)	269@10 mA cm^-2^	—	1.0 M KOH	Xie et al. (2025)
	90 (Thermoelectric)	240@10 mA cm^-2^	—	1.0 M KOH	Xie et al. (2025)
Fe_64_Ni_36_/FeNiO_x_H_y_	30 (Pure Electric)	245@10 mA cm^-2^	—	1.0 M KOH	Lu et al. (2024a)
	90 (Thermoelectric)	200@10 mA cm^-2^	—	1.0 M KOH	Lu et al. (2024a)
NiFeN@NiFeOOH	30 (Pure Electric)	320@10 mA cm^-2^	—	1.0 M KOH	Lu et al. (2022)
	90 (Thermoelectric)	220@10 mA cm^-2^	—	1.0 M KOH	Lu et al. (2022)
NiFeO_x_H_y_	30 (Pure Electric)	256@100 mA cm^-2^	31.4	1.0 M KOH	Liu et al. (2020)
	90 (Thermoelectric)	221@100 mA cm^-2^	—	1.0 M KOH	Liu et al. (2020)
cat.-51.9 (NiOOH)	25 (Pure Electric)	282.3@20 mA cm^-2^	47.8	1.0 M KOH	Liu et al. (2022)
	51.9 (Thermal Power)	∼1.5 V (Cell voltage) @10 mA cm^-2^	—	1.0 M KOH	Liu et al. (2022)
Sr_3_Fe_2_O_7_@SrFeOOH	30 (Pure Electric)	∼430@10 mA cm^-2^	—	1.0 M KOH	Lu et al. (2024b)
	90 (Thermoelectric)	∼245@10 mA cm^-2^	—	1.0 M KOH	Lu et al. (2024b)
Ni_0.9_Co_0.1_(OH)_2_ (E-TAC)	∼25 (Pure electric)	—	—	5.0 M KOH	Dotan et al. (2019)
	Step 2–95 (Thermoelectric)	1.44 V (Cell voltage) @10 mA cm^-2^	—	5.0 M KOH	Dotan et al. (2019)
Fe-MOF (S-treated)	25 (Pure Electric)	218@10 mA cm^-2^	36.2	1.0 M KOH	Feng et al. (2020)
	50 (Thermoelectric)	296@1000 mAcm^-2^	—	1.0 M KOH	Feng et al. (2020)

The integration of photothermal materials with electrocatalysts has also been explored for overall water splitting. The Co_9_S_8_@Ce-NiCo LDH/NF heterostructure achieved an overpotential of 144 mV for HER and 229 mV for OER at 100 mA cm^-2^ under NIR irradiation. The photothermal effect was attributed to the narrow bandgap of Co_9_S_8_, enabling efficient light-to-heat conversion, and the strategy was extended to other electrocatalysts such as NiCo-P, NiFeMo,and NiFe(OH)_x_, demonstrating the universality of Co_9_S_8_ as a photothermal promoter (Yang et al., 2024). P-doped NiFe_2_O_4_ electrocatalysts exhibited self-driving photothermal conversion under 808 nm NIR irradiation, with surface temperature increasing to 73 °C and overpotential at 10 mA cm^-2^ decreasing to 191 mV. The photothermal effect involved both thermal activation and hot carrier generation, with hot carriers altering the rate-determining step from O* to OOH* formation (Cai et al., 2024).

## 4 Challenges and perspectives

Thermo-electricity coupling has emerged as a highly effective strategy to boost water electrolysis by utilizing thermal energy beyond pure electrical input. The thermal fields could significantly regulate lattice strain, spin configurations, charge disproportionation and surface reconstruction of materials, enabling intrinsically enhanced OER/HER kinetics. Despite significant progress, there are still some challenges, such as the lack of deep understanding of the mechanism under actual coupling conditions, the development of low-cost and temperature-sensitive catalysts, the device level integration with waste heat recovery and long-term stability at high temperatures. Future research should focus on clarifying dynamic heat-electricity mechanisms via *in-situ*/operando characterizations, developing low-cost high-performance thermally responsive catalysts, designing compact heat-electricity integrated electrolyzers, and exploring multi-field coupling systems. Beyond the above fundamental challenges, several promising targeted research directions deserve deeper exploration to advance thermo-electric coupled water splitting toward industrialization. Firstly, the artificial intelligence (AI) and machine learning can be adopted to accelerate the development of thermally sensitive catalysts. For example, the multi-task predictive models trained on thermal strain, spin transition and OER activity datasets enabled rapid high-throughput screening of candidate materials, greatly reducing repeated trial and error synthesis and characterization costs. Besides, the digital twin technology could be constructed to realize full-process real-time simulation of coupled thermal-electric fields inside electrolyzers, dynamically predicting temperature gradients, interface charge transfer and long-term structural degradation for intelligent operation adjustment. Moreover, the dedicated reactor engineering optimization is required to achieve homogeneous temperature distribution, effective thermal insulation and maintained heat circulation within electrolytic cells, thereby eliminating local overheating and uneven reaction kinetics. Beyond that, it is promising that the scalable integration technology combining thermoelectric components with high-temperature electrolyzers should be further developed to realize low-grade industrial waste heat full recycling, so as to match large-scale green hydrogen production scenarios. These emerging technical paths is hopeful to complement material-level research and provide clear actionable research direction for subsequent experimental and engineering studies on thermal-field coupled water electrolysis.

## References

[B1] Abraham T. J. MacFarlane D. R. Pringle J. M. (2013). High seebeck coefficient redox ionic liquid electrolytes for thermal energy harvesting. Energy Environ. Sci. 6 (9), 2639–2645. 10.1039/C3EE41608A

[B2] Ai L. H. Tian Y. Xiao T. Y. Zhang J. Y. Zhang C. H. Jiang J. (2024). Photothermal RuNiFeO_x_/FeNi_3_ heterostructured arrays with Janus wettability for highly enhanced oxygen evolution reaction. Fuel 373, 132368. 10.1016/j.fuel.2024.132368

[B3] Cai J. J. Tang X. X. Wang J. M. Zhang T. T. Xie Q. Mao K. K. (2024). Self-driving photothermal anode electrocatalyst towards the robust OER for water electrolysis. Renew. Energy 232, 121121. 10.1016/j.renene.2024.121121

[B4] Dotan H. Landman A. Sheehan S. W. Malviya K. D. Shter G. E. Grave D. A. (2019). Decoupled hydrogen and oxygen evolution by a two-step electrochemical-chemical cycle for efficient overall water splitting. Nat. Energy 4 (9), 786–795. 10.1038/s41560-019-0462-7

[B5] Du Y. Xie F. K. Lu M. F. Lv R. X. Liu W. X. Yan Y. D. (2024). Continuous strain tuning of oxygen evolution catalysts with anisotropic thermal expansion. Nat. Commun. 15 (1), 1780. 10.1038/s41467-024-46216-9 38418515 PMC10901830

[B6] Fan X. Tan S. Y. Yang J. J. Liu Y. X. Bian W. Y. Liao F. (2022). From theory to experiment: cascading of thermocatalysis and electrolysis in oxygen evolution reactions. ACS Energy Lett. 7 (1), 343–348. 10.1021/acsenergylett.1c02380

[B7] Fan X. H. Fu Q. Liu G. R. Jia H. L. Dong X. L. Li Y. F. (2024). Applying molecular oxygen for organic pollutant degradation: strategies, mechanisms, and perspectives. Environ. Sci. Ecotechnol. 22, 100469. 10.1016/j.ese.2024.100469 39262838 PMC11387708

[B8] Feng K. Zhang D. Liu F. F. Li H. Xu J. B. Xia Y. J. (2020). Highly efficient oxygen evolution by a thermocatalytic process cascaded electrocatalysis over sulfur-treated Fe-based metal-organic-frameworks. Adv. Energy Mat. 10 (16), 2000184. 10.1002/aenm.202000184

[B9] Gao X. Q. Chen Y. T. Wang Y. J. Zhao L. Y. Zhao X. Y. Du J. (2024). Next-generation green hydrogen: progress and perspective from electricity, catalyst to electrolyte in electrocatalytic water splitting. Nano-Micro Lett. 16 (1), 237. 10.1007/s40820-024-01424-2 38967856 PMC11226619

[B10] Guo K. L. Wang Y. T. Yang S. Z. Huang J. F. Zou Z. H. Pan H. R. (2021). Bonding interface boosts the intrinsic activity and durability of NiSe@Fe_2_O_3_ heterogeneous electrocatalyst for water oxidation. Sci. Bull. 66 (1), 52–61. 10.1016/j.scib.2020.06.003 36654313

[B11] Guo L. J. Chen W. Wang C. Q. Dong B. R. (2023). Application of electrochemically assisted synthesis of MOFs-derived phosphides as catalyst for CH_4_-CO_2_ reforming. Int. J. Electrochem. Sci. 18 (1), 26–32. 10.1016/j.ijoes.2023.01.005

[B12] Hedayati D. P. Singh G. Schelkow R. Kucher M. Malik S. Keene T. D. (2026). Thermo-electrochemical coupled modeling of solid-state supercapacitors. Solid State Electrochem 30 (1), 41–55. 10.1007/s10008-025-06203-6

[B13] Hou Z. Q. Cui C. H. Li Y. N. Gao Y. J. Zhu D. M. Gu Y. F. (2023). Lattice-strain engineering for heterogenous electrocatalytic oxygen evolution reaction. Adv. Mat. 35 (39), 2209876. 10.1002/adma.202209876 36639855

[B14] Jabeen S. Saleem M. Mumtaz F. Javed S. Ali M. Khan M. Z. (2025). Coupling the power of spinel nanoparticles: dual-function metal ferrite catalysts for advanced piezo-photocatalytic and electrocatalytic water splitting. J. Mat. Chem. A 13 (36), 29911–29929. 10.1039/D5TA01180A

[B15] Jamesh M.-I. Hu D. Q. Wang J. Naz F. Feng J. P. Yu L. (2024). Recent advances in noble metal-free electrocatalysts to achieve efficient alkaline water splitting. J. Mat. Chem. A 12 (20), 11771–11820. 10.1039/D3TA07418H

[B16] Kazemi A. Manteghi F. Tehrani Z. (2024). Metal electrocatalysts for hydrogen production in water splitting. ACS Omega 9 (7), 7310–7335. 10.1021/acsomega.3c07911 38405471 PMC10882616

[B17] Li N. Luo X. L. Liu Z. Sun J. L. Zhou Q. L. Wang X. X. (2026). Ru nanocluster–mediated electronic modulation of NiFe-LDH for enhanced electrocatalytic water splitting. Carbon 247, 121033. 10.1016/j.carbon.2025.121033

[B18] Liu X. Guo R. T. Ni K. Xia F. J. Niu C. J. Wen B. (2020). Reconstruction-determined alkaline water electrolysis at industrial temperatures. Adv. Mat. 32 (40), 2001136. 10.1002/adma.202001136 32876959

[B19] Liu D. D. Lu M. F. Liu D. P. Yan S. C. Zhou W. Zhang L. Y. (2022). Heat-triggered ferri-to-paramagnetic transition accelerates redox couple-mediated electrocatalytic water oxidation. Adv. Funct. Mat. 32 (32), 2111234. 10.1002/adfm.202111234

[B20] Lu M. F. Li G. Q. Yan S. C. Zhang L. Y. Yu T. Zou Z. G. (2022). Heat-induced magnetic transition for water electrolysis on NiFeN@NiFeOOH core-shell assembly. Nano Lett. 22 (22), 9131–9137. 10.1021/acs.nanolett.2c03634 36317889

[B21] Lu M. F. Du Y. Yan S. C. Yu T. Zou Z. G. (2024a). Thermally stimulated spin switching accelerates water electrolysis. Phys. Rev. Lett. 133 (25), 258001. 10.1103/PhysRevLett.133.258001 39752681

[B22] Lu M. F. Du Y. Yan S. C. Yu T. Zou Z. G. (2024b). Thermal suppression of charge disproportionation accelerates interface electron transfer of water electrolysis. Proc. Natl. Acad. Sci., 121(1), e2316054120. 10.1073/pnas.2316054120 38147548 PMC10769854

[B23] Ma K. W. Chang X. R. Wang Z. H. Deng R. C. Wu X. Yang H. (2023). Tunable d-band center of a NiFeMo alloy with enlarged lattice strain enhancing the intrinsic catalytic activity for overall water-splitting. Nanoscale 15 (12), 5843–5854. 10.1039/D2NR07150A 36861662

[B24] Matienzo D. D. Kutlusoy T. Divanis S. Bari C. D. Instuli E. (2020). Benchmarking perovskite electrocatalysts’ OER activity as candidate materials for industrial alkaline water electrolysis. Catalysts 10 (12), 1387. 10.3390/catal10121387

[B25] McCrory C. C. L. Jung S. Peters J. C. Jaramillo T. F. (2013). Benchmarking heterogeneous electrocatalysts for the oxygen evolution reaction. J. Am. Chem. Soc. 45, 16977–16987. 10.1021/ja407115p 24171402

[B26] Mumtaz F. Jabbar H. Khan M. Z. Ghaffar A. Baluch A. H. Javed S. (2024). Optimization of z-scheme Bi_0.5_Na_0.5_TiO_3_/RGO-Co_3_O_4_ composite catalyst for water splitting reaction through piezo-photocatalysis. Int. J. Hydrogen Energy 78, 1468–1480. 10.1016/j.ijhydene.2024.06.387

[B27] Ou J. H. Yu S. Xie Y. Y. Jiang H. Lyu C. Xia G. Z. (2025). Pre-strain and release in MOF catalysts: Ligand-Engineered strain storage for enhanced catalytic activity. J. Colloid Interface Sci. 700, 138325. 10.1016/j.jcis.2025.138325 40628055

[B28] Qaseem A. Chen F. Y. Shabbir A. Saleem M. Koh J. H. Khan M. Z. (2026). Interfacially coupled and synergistic effect of Ag/Co_3_O_4_-C nanocomposites for enhanced oxygen reduction (ORR) and evolution (OER) reaction. Adv. Sci. 13 (14), e20911. 10.1002/advs.202520911 41588743 PMC12970163

[B29] Seh Z. W. Kibsgaard J. Dickens C. F. Chorkendorff I. Nørskov J. K. Jaramillo T. F. (2017). Combining theory and experiment in electrocatalysis: insights into materials design. Science 355 (6321), eaad4998. 10.1126/science.aad4998 28082532

[B30] Suen N. T. Hung S.-F. Quan Q. Zhang N. Xu Y. J. Chen H. M. (2017). Electrocatalysis for the oxygen evolution reaction: recent development and future perspectives. Chem. Soc. Rev. 46 (2), 337–365. 10.1039/C6CS00328A 28083578

[B31] Sun S. M. Wang W. Z. Jiang D. Zhang L. Zhou J. (2014). Infrared light induced photoelectrocatalytic application via graphene oxide coated thermoelectric device. Appl. Catal. B 158-159, 136–139. 10.1016/j.apcatb.2014.04.009

[B32] Tian G. Zhang C. X. Wei F. (2024). Fueling the future: innovating the path to carbon-neutral skies with CO_2_-to-aviation fuel. Carbon Future 1 (2), 9200010. 10.26599/CF.2024.9200010

[B33] Ul Sahar K. Rafiq K. Ur Rehman U. Hussain E. (2026). Frontiers in electrocatalytic water splitting: mechanistic pathways, catalytic engineering, and kinetic challenges. J. Energy Chem. 113, 692–737. 10.1016/j.jechem.2025.09.038

[B34] Wu L. B. Lu W. H. Ong W. L. Wong A. S. W. Zhang Y. M. Zhang T. X. (2025). Photothermal-promoted anion exchange membrane seawater electrolysis on a nickel-molybdenum-based catalyst. Nat. Commun. 16 (1), 3098. 10.1038/s41467-025-58320-5 40164589 PMC11958654

[B35] Xie F. K. Du Y. Lu M. F. Yan S. C. Zou Z. G. (2025). Thermal-stimulated spin disordering accelerates water electrolysis. Energy Environ. Sci. 18 (4), 1972–1983. 10.1039/D4EE04597A

[B36] Xiong Y. He P. (2023). A review on electrocatalysis for alkaline oxygen evolution reaction (OER) by Fe-based catalysts. J. Mat. Sci. 58 (5), 2041–2067. 10.1007/s10853-023-08176-1

[B37] Yan H. H. Jiang Z. Q. Deng B. L. Wang Y. J. Jiang Z. J. (2023). Ultrathin carbon coating and defect engineering promote RuO_2_ as an efficient catalyst for acidic oxygen evolution reaction with super-high durability. Adv. Energy Mat. 13 (23), 2300152. 10.1002/aenm.202300152

[B38] Yang L. Wang M. X. Shan H. Ma Y. M. Peng Y. J. Hu K. H. (2024). Generic heterostructure interfaces bound to Co_9_S_8_ for efficient overall water splitting supported by photothermal. J. Colloid Interface Sci. 662, 748–759. 10.1016/j.jcis.2024.02.126 38377694

[B39] Zhang Y. Q. Liu M. Zhang L. T. Lu N. Wang X. M. Li Z. G. (2025a). Strategic defect engineering enabled efficient oxygen evolution reaction in reconstructed metal-organic frameworks. Adv. Funct. Mat. 35 (4), 2412406. 10.1002/adfm.202412406

[B40] Zhang Z. Y. Zhao H. Y. Xi S. B. Zhao X. X. Chi X. Bin Yang H. (2025b). Breaking linear scaling relationships in oxygen evolution via dynamic structural regulation of active sites. Nat. Commun. 16 (1), 1301. 10.1038/s41467-024-55150-9 39900893 PMC11790916

[B41] Zhao L. L. Yang Z. Y. Cao Q. Yang L. J. Zhang X. F. Jia J. (2019). An earth-abundant and multifunctional Ni nanosheets array as electrocatalysts and heat absorption layer integrated thermoelectric device for overall water splitting. Nano Energ 56, 563–570. 10.1016/j.nanoen.2018.11.035

[B42] Zhou Y. Y. Ma Y. B. Wang X. Y. Yang G. Y. Ding Y. Q. Cui Y. S. (2023). Leaf-structure-inspired through-hole electrode with boosted mass transfer and photothermal effect for oxygen evolution reactions. Adv. Funct. Mat. 33 (43), 2304296. 10.1002/adfm.202304296

[B43] Zhou Q. L. Liu Z. Wang X. X. Li Y. Q. Qin X. Guo L. J. (2024). Co_3_S_4_-pyrolysis lotus fiber flexible textile as a hybrid electrocatalyst for overall water splitting. J. Energy Chem. 89, 336–344. 10.1016/j.jechem.2023.10.015

